# ﻿*Epimediumlongnanense* (Berberidaceae), a new species from Gansu, China

**DOI:** 10.3897/phytokeys.219.94275

**Published:** 2023-02-08

**Authors:** Jianhang Jiang, Ying Ji, Jianqiang Li, Yanjun Zhang

**Affiliations:** 1 Key Laboratory of Plant Germplasm Enhancement and Specialty Agriculture, Wuhan Botanical Garden, Chinese Academy of Sciences, Wuhan 430074, Hubei, China Wuhan Botanical Garden, Chinese Academy of Sciences Wuhan China; 2 Shangzhi Nature Studio of Shaanxi Tianyuan Chinese Herbal Medicine Development Co., Ltd., Mei County, Shaanxi Province, China Shangzhi Nature Studio of Shaanxi Tianyuan Chinese Herbal Medicine Development Co., Ltd. Meixian China

**Keywords:** *Epimedium*, IUCN Red List, morphology, taxonomy

## Abstract

*Epimediumlongnanense*, a new species of *Epimedium* (Berberidaceae) from Longnan Prefecture, Gansu Province, China, is here proposed and illustrated. *E.longnanense* has large flowers with petals possessing long spur and obvious basal lamina, and thus should be grouped into series *Davidianae*. The species closely resembles *E.flavum* of ser. Davidianae in morphology. However, it can be easily distinguished by its elongated rhizome (vs. compact), trifoliolate leaves (vs. five leaflets, sometimes trifoliolate), pale pink or purplish-red inner sepals with 6–8 × 2–3 mm (vs. pale sulphur-yellow, ca. 11 × 4 mm).

## ﻿Introduction

*Epimedium* Linnaeus, the largest herbaceous genus of Berberidaceae, is a relatively primitive group of angiosperms (Stearn 2002; Zhang et al. 2007). Currently, there are about 62 known species of *Epimedium* (Zhang et al. 2022b). They are widely and intermittently distributed in a narrow strip between Japan in Asia and Algeria in northern Africa (Ying 2002). With about 52 species, China is the center of diversity and distribution for *Epimedium* (Ying et al. 2011; Zhang et al. 2022b). *Epimedium* plants are excellent horticultural crops due to their beautiful and diverse leaves and flowers (Li et al. 2020). *Epimedium* has also been reported to have important medicinal values, such as strengthening the kidneys, curing rheumatism, and helping to fight osteoporosis, and tumors (Zhang and Yang 2021; Zhao et al. 2022).

In June 2021, we collected an unusual plant of *Epimedium* from Kangnan Forest District, Longnan Prefecture, Gansu province, China. In June 2022, we went to the forest district on two further occasions to investigate this plant of *Epimedium*. The plant is a low-growing herb with a height of 15–20 cm, bearing slender and elongated rhizomes. Its leaves are trifoliolate, and the leaflets are broadly ovate or almost orbicular and relatively small (2.4–4 × 2.3–4 cm). Furthermore, the plant possesses large yellow flowers and its petals are long spurred with an obvious basal lamina, and thus should be a member of ser. Davidianae Stearn. However, this plant has obvious morphological differences from other species of ser. Davidianae by its elongated rhizome, leaflet number, leaflet morphology, leaf arrangement on the flowering stem, flower color, and shape and size of inner sepals and petals. We confirmed that the plant should be a new species, which we describe below.

## ﻿Materials and methods

We have been engaged in taxonomic research of Chinese *Epimedium* since 2004. We examined all the specimens of *Epimedium* from the main herbaria of China (BCMM, CDCM, CDBI, GXMI, GZTM, HGAS, HGCM, HIB, HNNU, HNWP, HWA, IBK, IMD, KUN, PE, SAU, SM and SZ). Other *Epimedium* herbaria of China were examined from the Chinese virtual herbarium (http://www.cvh.ac.cn/). We also investigated the images of specimens of *Epimedium* from K, P, WU and WUK obtained by email or their network databases. In total, about 1700 *Epimedium* specimens have been intensively studied. Furthermore, our field investigations covered most of the distribution regions of Chinese *Epimedium*, including the following provinces, Anhui, Chongqing, Gansu, Guangdong, Guangxi, Guizhou, Henan, Hubei, Hunan, Jiangxi, Jilin, Liaoning, Shanxi, Shaanxi, Sichuan and Zhejiang of China. Almost all of the Chinese species of *Epimedium* were collected and transplanted to Wuhan Botanical Garden, the Chinese Academy of Sciences, for further study and conservation. For the new species of *Epimedium*, we compared its morphological characters with other species of the genus based on the specimen review and cultivation observation, as well as investigating its distribution and habitat. The morphological characters were described using the terminology used by Stearn (2002), Ying et al. (2011) and Zhang et al. (2022b).

## ﻿Resulsts

### ﻿ Epimedium longnanense

Taxon classificationPlantaeRanunculalesBerberidaceae

Y.J.Zhang sp. nov.

B8743784-7ADB-57E5-A940-077242F75D9F

urn:lsid:ipni.org:names:77313386-1

Figs 1 , 2

#### Type.

China, Gansu, Longnan Prefecture, Kangxian County, Kangnan Forest District, 32°59'N, 105°38'E, alt. 2000 m, 15 June 2022, *Yanjun Zhang 709* (holotype, HIB!; isotypes, HIB!).

**Figure 1.**
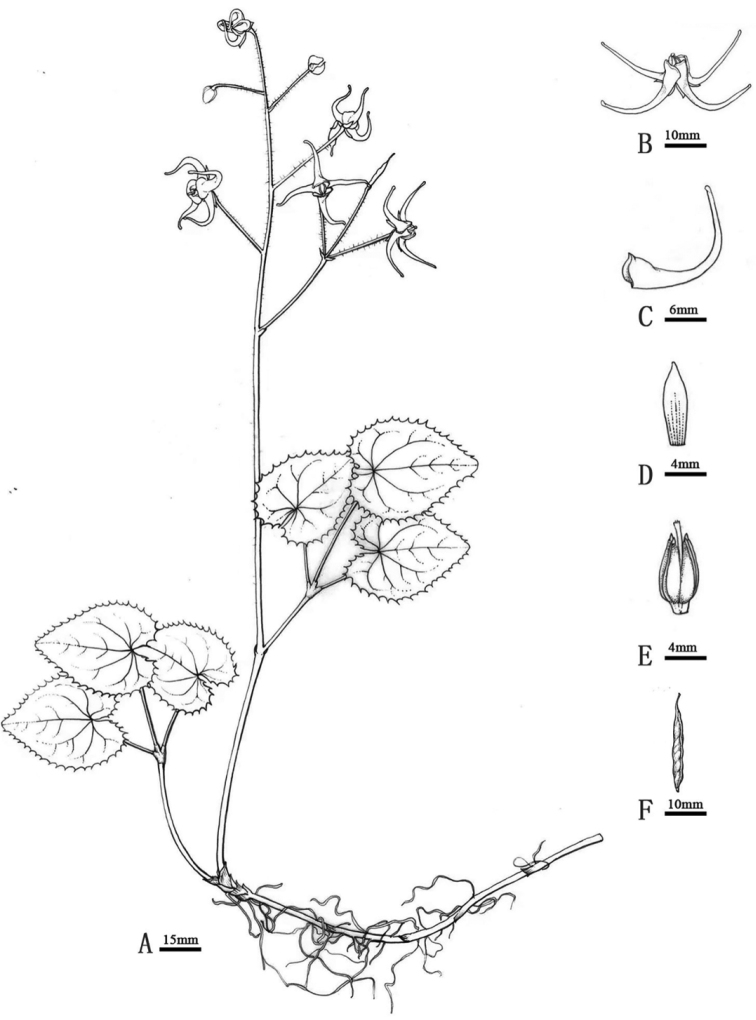
*Epimediumlongnanense***A** plant **B** flower **C** petal **D** inner sepal **E** stamen and gynoecium. Drawn by Nan Jia.

#### Diagnosis.

*Epimediumlongnanense* is closely similar to *E.flavum* Stearn in morphology, but can be easily distinguished by its elongated rhizome, trifoliolate leaves, and relatively smaller and pale pink or purplish-red inner sepals.

**Figure 2.**
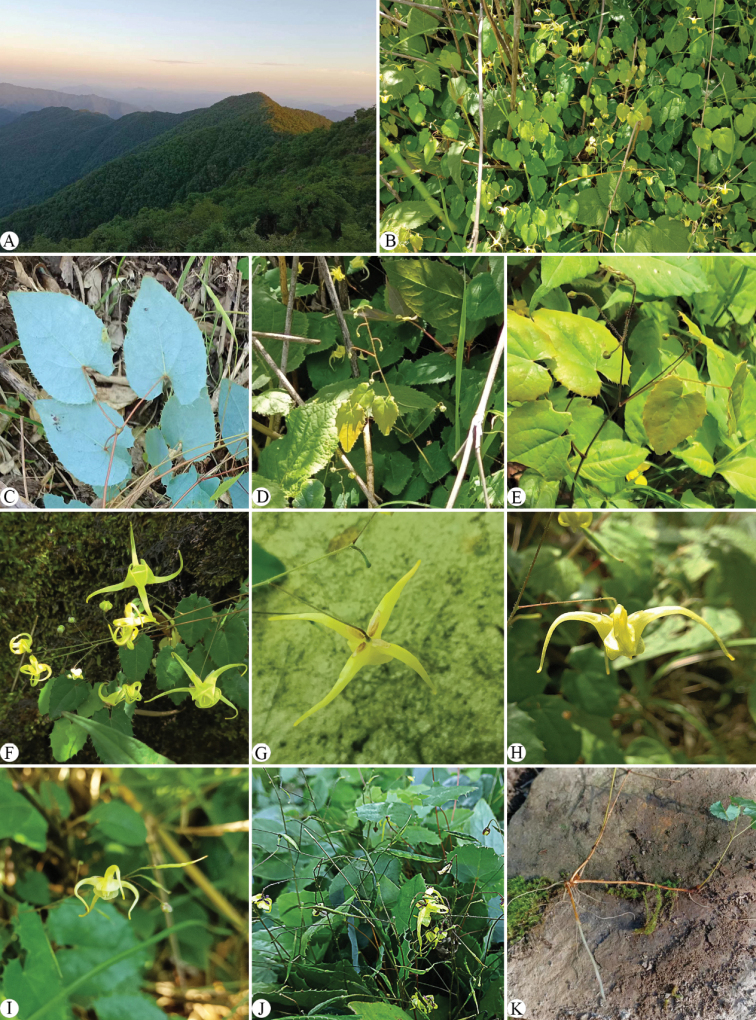
*Epimediumlongnanense***A** habitat **B** plant **C** leaves (abaxial sides) **D** simple inflorescence with a flowering stem bearing one leaf **E** compound inflorescence with a flowering stem bearing two alternative leaves **F** flowers (adaxial view) **G** flower (abaxial view) **H** flower (Side view) **I** flower (Side view) and immature capsules **J** mature capsules **K** rhizome. Photographed by Yanjun Zhang and Ying Ji.

#### Description.

Perennial herbs 15–20 cm tall. Rhizome ca. 15 cm or longer, ca. 1 mm in diam., elongated. Leaves basal and cauline, trifoliolate. Leaflets 2.4–4 × 2.3–4 cm, broadly ovate or almost orbicular, membranous, adaxially glabrous, abaxially sparsely pubescent, base deeply cordate with usually rounded lobes, those of lateral leaflets unequal, margins spinous-serrulate, apex usually acute or obtuse. Flowering stem with one leaf, or occasionally two alternate leaves, glabrous. Inflorescence compound or simple, 5–15-flowered, 10–15 cm long, glandular, pedicels 1.5–3.5 cm long. Flowers 3–5 cm in diam., yellow. Outer sepals ovate, soon falling; inner sepals 6–8 × 2–3 mm, narrowly ovate, pale pink or purplish-red. Petals 1.5–2.5 cm, much longer than inner sepals, horn-shaped, yellow, spurs slender; basal lamina 6–8 mm high, distinct. Stamens ca. 4 mm long, included, anthers ca. 3 mm long, anthers and pollen pale yellow. Capsules ca. 2 cm long.

#### Phenology.

Flowering in June; fruiting in late June and early July.

#### Etymology.

Longnan is located in the southeast of Gansu Province, the intersection of Qinba Mountain, Loess Plateau and Qinghai Tibet Plateau in China. Longnan is a biodiversity hotspot where many new species or new species records have been found (Liu et al. 2018; Qin et al. 2020; Zhang et al. 2022a). The new species, *E.longnanense*, is found in this region and is named after the geographic name.

#### Distribution and habit.

At present, *Epimediumlongnanense* is only known from its type locality, i.e. Kangnan Forest District, Kangxian County, Longnan Prefecture, Gansu Province, China. The new species mainly occurs in thickets at elevations of 1800–2300 m and is usually together with *Fargesianitida* (Mitford) Keng f. ex Yi, *Rubiacordifolia* L., *Smilaxstans* Maxim., *Cardaminetangutorum* O. E. Schulz and *Veratrumnigrum* L. It often grows in stone crevices with barren soil, and its elongated and slender rhizome might be an adaptive characteristic of its living environment. Except for *E.longnanense*, there are two other species of *Epimedium*, *E.brevicornu* Maxim. and *E.pubescens* Maxim., in Gansu Province, China. In the type locality of *E.longnanense*, we also found *E.pubescens* at elevations of 300–1500 m, but no sympatric distribution of these two species was found.

#### Chinese name.

Long nan yin yang huo (陇南淫羊藿).

#### Additional specimens examined (Paratypes).

China, Gansu Province, Longnan Prefecture, Kangxian County, Kangnan Forest District, alt. 2270 m, 20 June 2021, *Yanjun Zhang 699* (HIB!); loc. cit., alt. 1900 m, 30 June 2022, *Yanjun Zhang 710* (HIB!).

#### IUCN Red list category.

Data available for the new species are still insufficient to assess its conservation status. According to the IUCN criteria (IUCN 2022), it is considered Data Deficient (DD) until more information becomes available. Although *E.longnanense* currently has relatively good growth and protection status, we would like to elaborate that many other species of *Epimedium* have severely suffered from destructive excavation due to their huge medicinal values. Therefore, special attention should be given to the conservation of the new species of *Epimedium*.

### ﻿Key to species of Epimediumser.Davidianae

**Table:** 

1	Leaves biternate, or leaflets 7, 5, 3	**2**
–	Leaflets 3	**4**
2	Inner sepals pale sulphur-yellow, ca. 11 × 4 mm	**1. *E.flavum* Stearn**
–	Inner sepals purple red, 4–6.5 mm long	**3**
3	Leaves biternate, or leaflets 7, 5, rarely 3; inner sepals ovate, 5.5–6.5 × ca. 3 mm; petal spurs horizontally spreading with basal lamina 6–7 mm high	**2. *E.xichangense* Y. J. Zhang**
–	Leaflets 5, or 3; inner sepals narrowly ovate, ca. 4 × 1 mm; petal spurs downward-curved with basal lamina 7–13 mm high	**3. *E.davidii* Franch.**
4	Inflorescences racemose or compound	**5**
–	Inflorescences paniculate	**13**
5	Rhizome long-creeping, 1–3 mm in diam	**6**
–	Rhizome compact.	**10**
6	Leaflets broadly ovate or almost orbicular.	**7**
–	Leaflets ovate or narrowly ovate	**8**
7	Inflorescences simple, 3–4-flowered, flowers white, inner sepals 8–10 × ca.2.5 mm, spurs of petals ca. 1.2 cm long	**4. *E.pauciflorum* K. C. Yen**
–	Inflorescences compound or sometimes simple, 5–15-flowered, flowers yellow, inner sepals 6–8 × 2–3 mm, spurs of petals much longer than inner sepals, 1.5–2.5 cm long	**5. *E.longnanense* Y. J. Zhang**
8	Leaflets ovate, 4–5.5 × 2–2.5 cm	**6. *E.shuichengense* S. Z. He**
–	Leaflets narrowly ovate, 4–8 × 2–5.5 cm.	**9**
9	Inner sepals white, ovate, ca. 13 × 9 mm; petals purple, spurs a little longer than inner sepals, 15–16 mm long	**7. *E.epsteinii* Stearn**
–	Inner sepals reddish, cymbiform, ca. 6 × 2.5 mm; petals pale yellow, spurs much longer than inner sepals, ca. 22 mm long	**8. *E.fangii* Stearn**
10	Inner sepals white, petals white	**11**
–	Inner sepals white or pale pinkish-lilac, petals purple or chestnut-brown	**12**
11	Inner sepals lanceolate, 16–19 × 7–8 mm, apex acuminate; petals white, spurs almost as long as inner sepals, 15–18 mm long	**9. *E.ogisui* Stearn**
–	Inner sepals elliptic, ca. 16 × 8–9 mm, apex shortly acuminate; petals as long as inner sepals	**10. *E.latisepalum* Stearn**
12	Inner sepals narrowly ovate, white, petals deep purple, slightly shorter, or nearly as long as inner sepals	**11. *E.shengnongjiaense* Y. J. Zhang & J. Q. Li**
–	Inner sepals ovate, pale pinkish-lilac, petals chestnut-brown, a little longer than inner sepal, inner sepals pale pinkish-lilac	**12. *E.stearnii* Ogisu & Rix**
13	Petals with obvious basal lamina 7–8 mm high	**14**
–	Petals with slight base lamina 2–3.5 mm high	**15**
14	Leaflets oblong-elliptic or narrowly ovate, 10–13 × ca. 6 cm; inner sepals broadly elliptic, 5–6 × 3–4 mm	**13. *E.hunanense* (Hand.-Mazz.) Hand.-Mazz.**
–	Leaflets lanceolate or narrowly lanceolate, 9–23 × 1.8–4.5 cm; inner sepals ovate, ca. 12 × 6–8 cm	**14. *E.wushanense* T. S. Ying**
15	Inner sepals ovate or broadly ovate, 8–13 × 4–8 mm; spurs of petals slightly longer than inner sepals, 10–15 mm long	**15. *E.pseudowushanense* B. L. Guo**
–	Inner sepals ovate, 11–12 × 4–5.5 mm; spurs of petals obviously longer than inner sepals, 17–20 mm long	**16. *E.mikinorii* Stearn**

## ﻿Discussion

The floral trait is one of the most stable taxonomic characters in *Epimedium*, which has abundant diversities and is the focus of evolution studies of the genus (Ying 2002; Zhang et al. 2014; Guo et al. 2022). In the updated taxonomic system of *Epimedium* (Stearn 2002), all species endemic to China were classified into section Diphyllon Stearn of subgenus Epimedium Stearn. The section was divided into four series according to the flower morphology:ser. Campanulatae Stearn is characterized by campanulate flowers and flat petals without spur; ser. Davidianae Stearn bears large flowers and long spurred petals with obvious basal lamina; ser. Dolichocerae Stearn possesses large flowers and long spurred petals with no basal lamina; and ser. Brachycerae Stearn is characterized by short and spurred or saccate petals that are much shorter than the inner sepals. *Epimediumlongnanense* has large flowers with petals possessing long spur and obvious basal lamina, and thus should be grouped into ser. Davidianae.

So far, in total, there are 16 species in ser. Davidianae (Stearn 2002; Guo et al. 2007; Zhang and Li 2009; Ying et al. 2011; Zhang et al. 2016, 2022b). Among these 16 species of Epimediumser.Davidianae, *E.longnanense* is the most closely similar to *E.flavum* Stearn in morphology. However, *E.longnanense* can be easily distinguished by its rhizome, leaf and flower characters (Table 1). *E.longnanense* has a slender and elongated rhizome ca. 1 mm in diameter, while *E.flavum* has a compact rhizome 2–4 mm in diameter. Although their leaflets are similar in shape and size, *E.longnanense* has trifoliolate leaves, and *E.flavum* bears leaves usually with five leaflets, sometimes three leaflets. Furthermore, *E.longnanense* has smaller and pale pink or purplish-red inner sepals (6–8 × 2–3 mm), and its petals have spurs much longer than inner sepals (1.5–2.5 cm), while *E.flavum* has pale sulphur-yellow and larger sepals (ca. 11 × 4 mm), and its spurred petals are slightly longer than its inner sepals (ca. 1.3 cm).

**Table 1.** Comparison of morphological characters of *E.longnanense*, *E.flavum* and *E.membranaceum*.

Characters	*E.longnanense*	*E.flavum*	*E.membranaceum*
Plant height	15–20 cm	13–30 cm	20–65 cm
Rhizome	Elongated, ca. 1 mm	Compact, 2–4 mm	Compact or occasionally elongated, 2–4 mm
Number of leaflets	3	(3–)5	3
Leaves on the flowering stem	1 leaf or occasionally 2 alternate leaves	1 leaf or 2 opposite or alternate leaves	2 opposite or sometimes alternate leaves
Leaflets	Broadly ovate or almost orbicular, the tip acute or obtuse, 3.5–5.5 × 2.5–4.5 cm	Broadly ovate, the tip obtuse, ca. 4 × 3 cm	Broadly ovate or narrowly ovate, the tip acute or short-acuminate, 3–10 × 2–6 cm
Inflorescence	Compound or simple, 10–15 cm, 5–15-flowered	Simple or compound, 13 cm, 3–10-flowered	Compound, 16–30 cm, 5–35-flowered
Inner sepals	Pale pink or purplish-red, 6–8 × 2–3 mm	Pale sulphur-yellow, ca. 11 × 4 mm	Pale pink, 6–7 × 2.5–3 mm
Spur length of petals	1.5–2.5 cm	ca. 1.3 cm	1.5–2.5 cm
Basal lamina height of petals	6–8 mm	8 cm	–

Among the other three series of Chinese Epimediumsect.Diphyllon, *E.longnanense* closely resembles *E.membranaceum* in morphology, a species in ser. Dolichocerae. *Epimediummembranaceum* was treated as a synonym of *E.davidii* Franchet in Flora Reipublicae Popularis Sinica (Ying 2001) and Flora of China (Ying et al. 2011). However, *E.membranaceum* and *E.davidii* are two distinct species with obvious differences in leaflet number and floral characters (Stearn 2002; Zhang et al. 2015). Both *E.longnanense* and *E.membranaceum* have large yellow flowers, and their petals possess long, slender and slightly-curved spurs. However, *E.longnanense* is only 15–20 cm high with shorter inflorescence (10–15 cm), while *E.membranaceum* is taller (20–65 cm) with longer inflorescence (16–30 cm). Furthermore, *E.longnanense* has petals possessing an obvious basal lamina and is a member of ser. Davidianae, while *E.membranaceum* has petals with no lamina and is a member of ser. Dolichocerae (Table 1).

## Supplementary Material

XML Treatment for Epimedium longnanense
